# Supplementary material for: Matching sensor ontologies through siamese neural networks without using reference alignment
Source: PeerJ Comput Sci. 2021 Jun 18;7:e602. doi: 10.7717/peerj-cs.602 (PMC8237319; doi:10.7717/peerj-cs.602)
Supplement: Supplemental Information 1 [file peerj-cs-07-602-s001.zip › 304/onto.html]

bibtex ontology


# bibtex ontology

Transformation of bibTeX into an OWL ontology

*Possible ontology to describe bibTeX entries.*  
Author: Nick Knouf <nknouf@mit.edu>  
Contributor: Antoine Zimmermann <antoine.zimmermann@inrialpes.fr>,   
Date: 2004-01-10  
Version: 0.01

## Classes

**Entry** (Entry, *Base class for all entries)*
:   - #date [0 1]
    - #title [0 1]
    - #author [0 1]

    **Published** (Published, *.)*
    :   **Article** (Article, *An article from a journal or magazine.)*
        :   super: *#Part*  

            - #author [1 1]
            - #title [1 1]
            - #inJournal [1 1]
            - #date [1 1] *#DateWithMonth*
            - #number [0 1]
            - #volume [0 1]

        **Book** (Book, *A single book that may be a monography or a collection of written texts.)*
        :   super: *#Composite*  

            - #humanCreator [1 1]
            - #edition [0 1]

            **Monography** (Monography, *A book that is a single entity, as opposed to a collection.)*


            **Collection** (Collection, *A book that is collection of texts or articles.)*

        **BookPart** (BookPart, *May be a chapter (or section or whatever), a range of pages or a part of a book having its own title.)*
        :   super: *#Part*  

            - #chapter [0 1]
            - #pagesOrChapter [0 2]

    **Composite** (Book, *A work that may be composed of several parts or chapter. May be a monography, a collection or a proceedings.)*
    :   - #numberOrVolume [0 1]
        - #publisher [0 1]
        - #series [0 1]
        - #title [1 1] [1 1]
        - #date [1 1] *#DateWithMonth*

        **Book** (Book, *A single book that may be a monography or a collection of written texts.)*
        :   super: *#Published*  

            - #humanCreator [1 1]
            - #edition [0 1]

            **Monography** (Monography, *A book that is a single entity, as opposed to a collection.)*


            **Collection** (Collection, *A book that is collection of texts or articles.)*

        **Proceedings** (Proceedings, *The proceedings of a conference.)*
        :   - #publisher [0 1]
            - #editor [0 1]
            - #organization [0 1]

    **Informal** (Informal, *A document that that was informally published or not published.)*
    :   **Booklet** (Booklet, *A work that is printed and bound, but without a named publisher or sponsoring institution.)*
        :   - #title [1 1]

        **Deliverable** (Conference, *TROUVER UNE DESCRIPTION POUR CA.)*


        **LectureNotes** (LectureNotes, *Lecture notes.)*
        :   super: *#Academic*

        **Manual** (Manual, *Technical documentation.)*
        :   - #organization [0 1]
            - #edition [0 1]
            - #title [1 1]

        **Misc** (Misc, *Use this type when nothing else fits.)*


        **TechReport** (TechReport, *A report published by a school or other institution, usually numbered within a series.)*
        :   - #author [1 1]
            - #title [1 1]
            - #institution [1 1]
            - #date [1 1] *#DateWithMonth*
            - #number [0 1]

        **Unpublished** (Unpublished, *A document having an author and title, but not formally published.)*
        :   - #author [1 1]
            - #title [1 1]
            - #note [1 +oo]

    **Part** (Part, *A part of something (either Book or Proceedings).)*
    :   - #pages [0 1]
        - #title [1 1]
        - #isPartOf [1 1]

        **Article** (Article, *An article from a journal or magazine.)*
        :   super: *#Published*  

            - #author [1 1]
            - #title [1 1]
            - #inJournal [1 1]
            - #date [1 1] *#DateWithMonth*
            - #number [0 1]
            - #volume [0 1]

        **BookPart** (BookPart, *May be a chapter (or section or whatever), a range of pages or a part of a book having its own title.)*
        :   super: *#Published*  

            - #chapter [0 1]
            - #pagesOrChapter [0 2]

        **InProceedings** (InProceedings, *An article in a conference proceedings.)*
        :   - #author [1 1]
            - #inProceedings [1 1]

    **Academic** (Academic, *A Master's or PhD thesis.)*
    :   - #author [1 1]
        - #title [1 1]
        - #school [1 1]

        **LectureNotes** (LectureNotes, *Lecture notes.)*
        :   super: *#Informal*

        **Thesis** (Thesis, *A thesis (either Master or PhD).)*
        :   - #date [1 1] *#DateWithMonth*

            **MastersThesis** (MastersThesis, *A Master's thesis.)*


            **PhdThesis** (PhdThesis, *A PhD thesis.)*

    **MotionPicture** (MotionPicture, *A film/movie/motion picture.)*

**Institution** (Institution, *An institution.)*


**School** (School, *A school or university.)*


**Journal** (Journal, *A journal.)*


**Organization** (Organization, *An organization.)*


**Publisher** (Publisher, *A publisher.)*


**Person** (Person, *A person.)*


**PersonList** (Person list, *A list of persons.)*
:   super: *http://www.w3.org/1999/02/22-rdf-syntax-ns#List*  

    - http://www.w3.org/1999/02/22-rdf-syntax-ns#first [1 1] *#Person*
    - http://www.w3.org/1999/02/22-rdf-syntax-ns#rest [1 1] (*#PersonList* |  {

      <rdf:List@ttp://www.w3.org/1999/02/22-rdf-syntax-ns#nil>
      } )

**http://www.w3.org/1999/02/22-rdf-syntax-ns#List** (, *)*


**PageRange** (PageRange, *A range of pages.)*
:   - #startPage [1 1]
    - #endPage [1 1]

**Date** (Date, *Date of a day, month or year.)*
:   - #year [1 1]
    - #month [0 1]

    **DateWithMonth** (Date, *Date of a day, month or year.)*
    :   - #month [1 1]
        - #day [0 1]

**Text** (Text, *A simple text with possibly other information such as language, etc.)*
:   **Title** (Title, *A title.)*

## Properties

**annote**: #Entry -> #Text *(An annotation. It is not used by the standard bibliography styles, but may be used by others that produce an annotated bibliography.)*


**edition**: (*#Book* | *#Manual*) -> #Text *(The edition of a book--for example, "Second". This should be an ordinal, and should have the first letter capitalized, as shown here; the standard styles convert to lower case when necessary.)*


**howPublished**: (*#Misc* | *#Booklet*) -> #Text *(How something strange has been published. The first word should be capitalized.)*


**isPartOf**: #Part -> \_ *(The document that contains a text or article.)*
:   **inJournal**: #Article -> #Journal *(A journal name. Abbreviations are provided for many journals; see the Local Guide)*


    **inMonography**: #InBook -> #Monography *(A reference to the book that contains the entry. The entry must be an InBook end the book a Monography.)*


    **inCollection**: #InCollection -> #Collection *(A reference to the book that contains the entry. The entry must be an Inbook or Incollection.)*


    **inProceedings**: #InProceedings -> #Proceedings *(A reference to the proceedings in which the entry appears. The entry must be an InProceedings.)*

**institution**: #TechReport -> #Institution *(The sponsoring institution of a technical report.)*


**date**: #Entry -> #Date *(The date when the work was published or, for an unpublished work, in which it was written.)*


**note**: #Entry -> #Text *(Any additional information that can help the reader. The first word should be capitalized.)*


**organization**: (*#Proceedings* | *#Manual*) -> #Organization *(The organization that sponsors a conference or that publishes a manual.)*


**publisher**: #Composite -> #Publisher *(The publisher's name.)*


**school**: (*#Thesis* | *#LectureNotes*) -> #School *(The name of the school where a thesis was written.)*


**series**: #Composite -> #Title *(The name of a series or set of books. When citing an entire book, the the title field gives its title and an optional series field gives the name of a series or multi-volume set in which the book is published.)*


**title**: #Entry -> #Title *(The work's title, typed as explained in the LaTeX book.)*


**type**: (*#Chapter* | *#TechReport* | *#Thesis*) -> #Text *(The type of a technical report--for example, "Research Note".)*


**affiliation**: #Entry -> #Text *(The authors affiliation.)*


**abstract**: #Entry -> #Text *(An abstract of the work.)*


**contents**: #Entry -> #Text *(A Table of Contents.)*


**copyright**: #Entry -> #Text *(Copyright information.)*


**isbn**: #Entry -> #Text *(The International Standard Book Number.)*


**issn**: #Entry -> #Text *(The International Standard Serial Number. Used to identify a journal.)*


**keywords**: #Entry -> #Text *(Key words used for searching or possibly for annotation.)*


**language**: #Entry -> #Text *(The language the document is in.)*


**location**: #Entry -> #Text *(A location associated with the entry, such as the city in which a conference took place.)*


**lccn**: #Entry -> #Text *(The Library of Congress Call Number.)*


**mrNumber**: #Entry -> #Text *(The Mathematical Reviews number.)*


**price**: #Entry -> #Text *(The price of the document.)*


**size**: #Entry -> #Text *(The physical dimensions of a work.)*


**url**: #Entry -> #Text *(The WWW Universal Resource Locator that points to the item being referenced. This often is used for technical reports to point to the ftp or web site where the postscript source of the report is located.)*


**humanCreator**: #Entry -> #PersonList *(A generic human creator category, necessary in order to contain both author and editor.)*
:   **author**: #Entry -> #PersonList *(The name(s) of the author(s), in the format described in the LaTeX book.)*


    **editor**: #Composite -> #PersonList *(Name(s) of editor(s), typed as indicated in the LaTeX book. If there is also an author field, then the editor field gives the editor of the book or collection in which the reference appears.)*


    **directors**: #MotionPicture -> #PersonList *(The list of persons who directed the motion picture.)*

**pagesOrChapter**: (*#Part*) -> \_ *(A generic property to hold page and/or chapter data.)*
:   **chapter**: #Chapter -> #Text *(A chapter (or section or whatever) number.)*


    **pages**: (*#Part*) -> #PageRange *(A range of page, with an upper and lower limit.)*

**http://www.w3.org/1999/02/22-rdf-syntax-ns#first**: http://www.w3.org/1999/02/22-rdf-syntax-ns#List -> \_ *()*


**http://www.w3.org/1999/02/22-rdf-syntax-ns#rest**: http://www.w3.org/1999/02/22-rdf-syntax-ns#List -> \_ *()*

**key** #Entry -> http://www.w3.org/2001/XMLSchema#string *(The key for a particular bibTeX entry. Note that the rdf:ID for each Entry instance could be the bibTeX key as well, possibly making this property redundant.)*


**numberOrVolume**(*#Article* | *#Composite*) -> \_ *(The number of a journal, magazine, technical report, or of a work in a series. An issue of a journal or magazine is usually identified by its volume and number; the organization that issues a technical report usually gives it a number; and sometimes books are given numbers in a named series.)*
:   **number**(*#Article* | *#Composite*) -> http://www.w3.org/2001/XMLSchema#string *(The number of a journal, magazine, technical report, or of a work in a series. An issue of a journal or magazine is usually identified by its volume and number; the organization that issues a technical report usually gives it a number; and sometimes books are given numbers in a named series.)*


    **volume**(*#Article* | *#Composite*) -> http://www.w3.org/2001/XMLSchema#nonNegativeInteger *(The volume of a journal or multivolume book.)*

**startPage** #PageRange -> http://www.w3.org/2001/XMLSchema#nonNegativeInteger *(The beginning of a range of pages.)*


**endPage** #PageRange -> http://www.w3.org/2001/XMLSchema#nonNegativeInteger *(The end of a range of pages.)*


**year** #Date -> http://www.w3.org/2001/XMLSchema#nonNegativeInteger *()*


**month** #Date -> http://www.w3.org/2001/XMLSchema#nonNegativeInteger *()*


**day** #DateWithMonth -> http://www.w3.org/2001/XMLSchema#nonNegativeInteger *()*


**lang** #Text -> http://www.w3.org/2001/XMLSchema#string *(The language in which the words are written.)*


**short** #Title -> http://www.w3.org/2001/XMLSchema#string *(A shorten version of the title.)*

## Individuals

---

Generated by OWL2HTML
